# Density functional theory study on the formation mechanism and electrical properties of two-dimensional electron gas in biaxial-strained LaGaO_3_/BaSnO_3_ heterostructure

**DOI:** 10.1038/s41598-024-60893-y

**Published:** 2024-05-04

**Authors:** Yuling Li, Yuxi Huang, Xiaohua Liu, Yaqin Wang, Le Yuan

**Affiliations:** 1 Key Laboratory of Fluid and Power Machinery, School of Material Science and Engineering, Xihua University, Chengdu, 610039 People’s Republic of China; 2 State Key Laboratory of Electronic Thin Films and Integrated Devices, University of Electronic Science and Technology of China, Chengdu, 610054 People’s Republic of China

**Keywords:** Electronic structure, Surfaces, interfaces and thin films, Electronic properties and materials

## Abstract

The two-dimensional electron gas (2DEG) in BaSnO_3_-based heterostructure (HS) has received tremendous attention in the electronic applications because of its excellent electron migration characteristic. We modeled the *n*-type (LaO)^+^/(SnO_2_)^0^ interface by depositing LaGaO_3_ film on the BaSnO_3_ substrate and explored strain effects on the critical thickness for forming 2DEG and electrical properties of LaGaO_3_/BaSnO_3_ HS system using first-principles electronic structure calculations. The results indicate that to form 2DEG in the unstrained LaGaO_3_/BaSnO_3_ HS system, a minimum thickness of approximately 4 unit cells of LaGaO_3_ film is necessary. An increased film thickness of LaGaO_3_ is required to form the 2DEG for -3%-biaxially-strained HS system and the critical thickness is 3 unit cells for 3%-baxially-strained HS system, which is caused by the strain-induced change of the electrostatic potential in LaGaO_3_ film. In addition, the biaxial strain plays an important role in tailoring the electrical properties of 2DEG in LaGaO_3_/BaSnO_3_ HS syestem. The interfacial charge carrier density, electron mobility and electrical conductivity can be optimized when a moderate tensile strain is applied on the BaSnO_3_ substrate in the *ab*-plane.

## Introduction

Recently, the two-dimensional electron gas (2DEG) at the *n*-type (LaO)^+^/(TiO_2_)^0^ interface in LaAlO_3_/SrTiO_3_ heterostructures (HS) system has attracted considerable attention due to its unique interfacial properties and promising applications in the next-generation nanoelectronics^1–4^. The typical explanation for the formation of 2DEG is the so-called “polar catastrophe” mechanism^5,6^. The LaAlO_3_ film is formed by alternating charged (LaO)^+^ and (AlO_2_)^−^ layers, while the SrTiO_3_ substrate is considered as stacks of neutral (SrO)^0^ and (TiO_2_)^0^ layers. The *n*-type (LaO)^+^/(TiO_2_)^0^ interface can be formed by growing LaAlO_3_ films on the SrTiO_3_ substrate. Then an electronic reconstruction occurs at the LaAlO_3_/SrTiO_3_ interface to compensate the polar discontinuity by migrating electrons from the interfacial polar (LaO)^+^ layer to the adjacent non-polar (TiO_2_)^0^ layers.

Although the LaAlO_3_/SrTiO_3_ HS system exhibits a high interfacial carrier density with 3.2 $\times$ 10^−14^ cm^−2^, the electron mobility is low (1 cm^2^V^−1^s^−1^) at room temperature (RT), which limits its application in the photoelectric devices^7,8^. This phenomenon is originated from that the conduction band bottom of SrTiO_3_ is composed of highly dispersed $d_{xy}$ orbitals and the electrons on these orbitals show high mobility at low temperature. While it consists of lowly dispersed $d_{xz}$/$d_{yz}$ orbitals at RT. The multi-band degeneracy leads to the inter-band transition scattering and stronger electron-phonon coupling effect, which reduces the electron mobility. To broaden the application of LaAlO_3_/SrTiO_3_ HS system at RT, some approaches are proposed to improve its interfacial electron mobility. For instance, defect engineering^9,10^, strain engineering^11,12^, and find other materials to replace the SrTiO_3_ channel material^13,14^. Z. Q. Liu et al.^9^ pesented that the interfacial carrier density of LaAlO_3_/SrTiO_3_ HS system at RT increases with the decrease of oxygen partial pressure, and the carrier mobility shows the opposite trend. Ariando et al.^12^ reported that the electron mobility of interfacial 2DEG in LaAlO_3_/SrTiO_3_ HS system is sensitive to the biaxial strain. The biaxial compressive strain decreases the electron mobility and increases the interfaical carrier density. The largest electron mobility is $<10$ cm^2^V^−1^s^−1^at RT. Zou et al.^13^ prepared a polar/polar perovskite oxide heterostructure, that is, LaTiO_3_/KTaO_3_ HS system with (LaO)^+^/(TaO_2_)^+^ interface, and found that this HS system exhibits high interfacial electron mobility of 21 cm^2^V^−1^s^−1^ at RT, which is higher than that of well-known LaAlO_3_/SrTiO_3_ HS system. Therefore, the performance of LaAlO_3_/SrTiO_3_ HS system can be effectively regulated by perovskite channel material KTaO_3_. The search for other channel materials with high electron mobility at RT to further improve the interfacial electron mobility of pervoskite-type HS system has become a research focus.

BaSnO_3_ film with Sn 5*s* orbitals at the bottom of conduction bands has considered as an ideal material for oxide transistor channel material due to its high electron mobility at RT. This is because that the *s* orbitals are less localized than *d* orbitals, which results in larger band dispersion and lower electron effective mass. Compared with traditional SrTiO_3_ films, BaSnO_3_ film with *s* orbitals at the conduction band bottoms has extremely high electron mobility at RT, with a value of 150 cm^2^V^−1^s^−1^^15^. Our group have explored the possibility of producing a high-mobility 2DEG in LaGaO_3_/BaSnO_3_ HS system using first-principles electronic structure calculations. This HS system presented twice larger electron mobility and enhanced interfacial conductivity compared to the prototype LaAlO_3_/SrTiO_3_ HS system^16^. Kookrin et al.^17^ combined experimental and theoretical approach to study the different electrical properties of perovskite-type LaInO_3_/BaSnO_3_ HS system on MgO and SrTiO_3_ substrates, which are well explained by the varying deep acceptor densities for the HS system on two different substrates. They also reported that when the thickness of LaScO_3_ (LaInO_3_) film is 12 unit cells, the interfacial charge density of HS system at RT is about 2.5 $\times$ 10^13^ cm^−2^, and the electron mobility is about 20-25 cm^2^V^−1^s^−1^, which is obviously higher than that of LaAlO_3_/SrTiO_3_ system. Moreover, with the increase of La concentration doped in BaSnO_3_, the interfacial electron density and mobility of HS system shows an increasing trend^18,19^. Aggoune et al.^20^ preliminally explored the formation mechanism of 2DEG and two-dimensional hole gas (2DHG) by regulating the polarity and thickness of LaInO_3_ film, as well as the interface structure using the first-principles calculation. However, the detailed formation mechanism for 2DEG needs further systematically studied. Particularly, the changes of electrical properties of the BaSnO_3_-based HS systems deposited on the substrates with different lattice parameters also need further investigation. This lattice mismatch between the HS system and substrate is easy to form growth strain, which is also usually induced by varying experimental preparation parameters and the uneven thermal diffusion during the heating/cooling process caused by the mismatch of thermal expansion coefficient between the HS system and substrate. Previous researchers^12,21^ have found that this growth strain is generally in the range of – 3% $\sim$ 3%. For example, Z. Huang et al.^12^ selected four substrates with different lattice parameters to prepare Nb-SrTiO_3_ thin film and LaAlO_3_/SrTiO_3_ HS system. The lattice mismatch between the HS system and substrate resulted in – 2.98% (LaAlO_3_), – 0.96% (LSAT), 0 (SrTiO_3_), and 0.99% (DyScO_3_) strains, respectively. C. W. Bark et al.^21^ deposited LaAlO_3_/SrTiO_3_ HS system on the substrates with different lattice parameters by pulsed laser deposition, resulting in a biaxial range of – 1.21% (NdGaO_3_) $\sim$ 1.59% (GdScO_3_) in the HS system. Lan Meng et al.^22^ prepared WS_2_ film by chemical vapor deposition on SiO_2_/Si substrate, and then cooled it rapidly. In the process of rapidly cooling, the mismatch of thermal expansion coefficient between WS_2_ film and substrate led to local stress in WS_2_ film, but the stress/strain value was not referred to in this paper. Besides growth strain, we can dynamically regulate the force of film/heterostructure by artificially applying strain in the experiments, which is called “extrinsic strain”. Then the range of strain can be artificially regulated according to the ultimate force of the material. For example, B Jalan et al.^23^ applied different stresses on the epitaxial SrTiO_3_ thin films by three-point bending, studying the changes of electron mobility of film with the temperature and stress.

**Figure 1:**
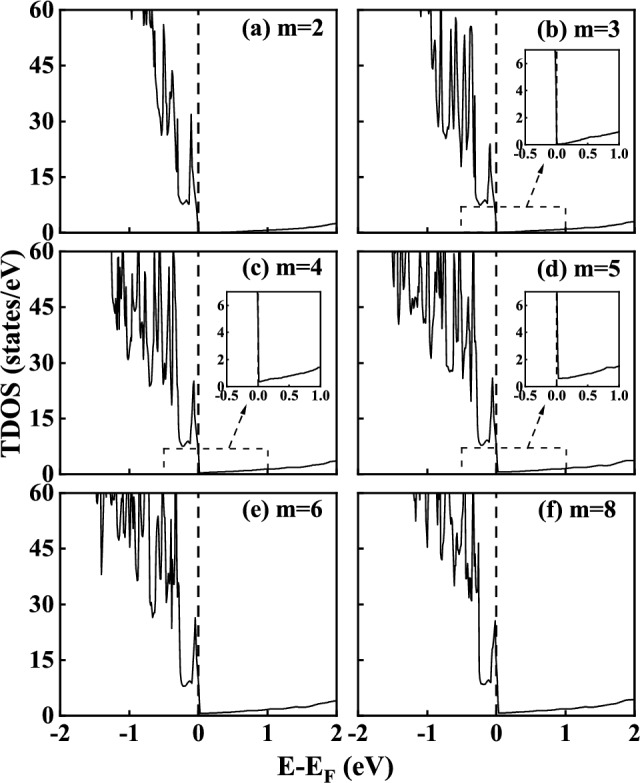
Calculated total density of states (DOS) for the *n*-type (LaGaO_3_)_*m*_/BaSnO_3_ HS models with different LaGaO_3_ unit cells. (**a**) m = 2, (**b**) m = 3, (**c**) m = 4, (**d**) m = 5, (**e**) m = 6 and (**f**) m = 8. The vertical dashed line indicates the Fermi level at 0 eV in this and each subsequent DOS plot. The insets are the enlarged view of the DOS near fermi level for models.

In this work, we systematically investigated the (LaGaO_3_)_*m*_/BaSnO_3_ HS models by means of the first-principles calculations. Firstly, the LaGaO_3_ film thickness dependence of the formation of 2DEG in the unstrained (LaGaO_3_)_*m*_/BaSnO_3_ HS model was studied. Next, we explored the influence of biaxial strain in the *ab*-plane on the critical thickness of LaGaO_3_ film for forming 2DEG. The electrical properties of LaGaO_3_/BaSnO_3_ HS system dependent on the biaxial strain were analyzed from the electron effective mass, interfacial electron density, electron mobility, and electrical conductivity. This work may provide some guidances for adjusting the electrical properties of LaGaO_3_/BaSnO_3_ HS system by biaxial strain.

## Results

### Bulk parent compounds

First, we calculated the lattice parameters and energy band gap of LaGaO_3_ and BaSnO_3_ materials in their cubic phase, shown in Table S1 in the supporting information. The calculated lattice constants from GGA+U functional are well consistent with the experimental values (3.939 vs 3.860 Åfor LaGaO_3_ and 4.186 vs 4.115 Åfor BaSnO_3_)^24,25^. In contrast, the calculated energy band gaps from the GGA+U approach are underestimated with respect to the experimental values (3.668 vs 4.4 eV for LaGaO_3_ and 2.208 vs 3.1 eV for BaSnO_3_)^26,27^, which is due to the well-known shortcoming of the GGA functional that cannot give an accurate description for the electron-electron correlation-exchange interaction. However, this underestimation has been determined that it has no influence on our conclusions about the 2DEG at the perovskite-type HS systems because the electronic states that contribute to the formation of DEG can be well reproduced from GGA+U calculations^28–30^. Then our GGA+U approach can well predict the 2DEG-related Sn 5*s* states as well as the critical thickness of the LaGaO_3_ for forming the 2DEG in the LaGaO_3_/BaSnO_3_ HS system.

### 2DEG in the *n*-type LaGaO_3_/BaSnO_3_ HS system

The calculated total density of states (DOS) for the *n*-type (LaGaO_3_)_*m*_/BaSnO_3_ HS system with different LaGaO_3_ unit cells (*m*=2, 3, 4, 5, 6 and 8) are shown in Fig. 1, where the vertical dotted line at 0 eV represents the fermi level. At m=2 and 3, the fermi level of (LaGaO_3_)_*m*_/BaSnO_3_ HS system is on the top of the valence bands, and the HS system shows insulating characteristics. The band gap of HS system decreases with the increase of LaGaO_3_ film thickness. While at m=4, 5, 6 and 8, the band gap disappears and all the HS models exhibit metallic properties. With the increase of LaGaO_3_ film thickness, the metallic states near the fermi level increase. These results indicate that the critical thickness of insulator-to-metal transition is 4 unit cells for LaGaO_3_ film in the *n*-type LaGaO_3_/BaSnO_3_ HS system, which is similar to the case of well-known *n*-type LaAlO_3_/SrTiO_3_ HS model^31^.

**Figure 2:**
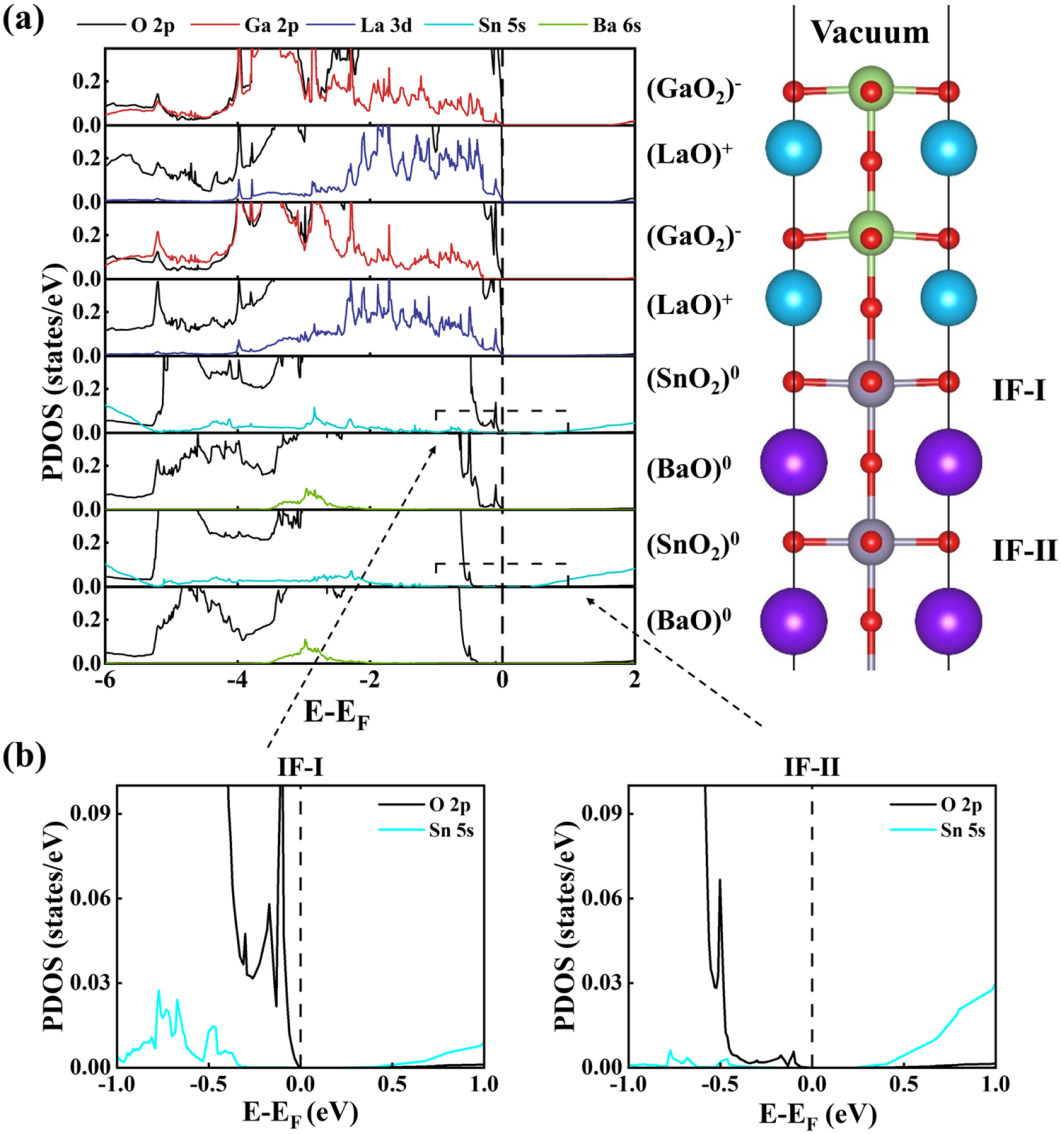
Calculated layer-resolved partial DOS for the *n*-type (LaGaO_3_)_2_/BaSnO_3_ HS model along with the charge density projected on bands forming the 2DEG.

**Figure 3:**
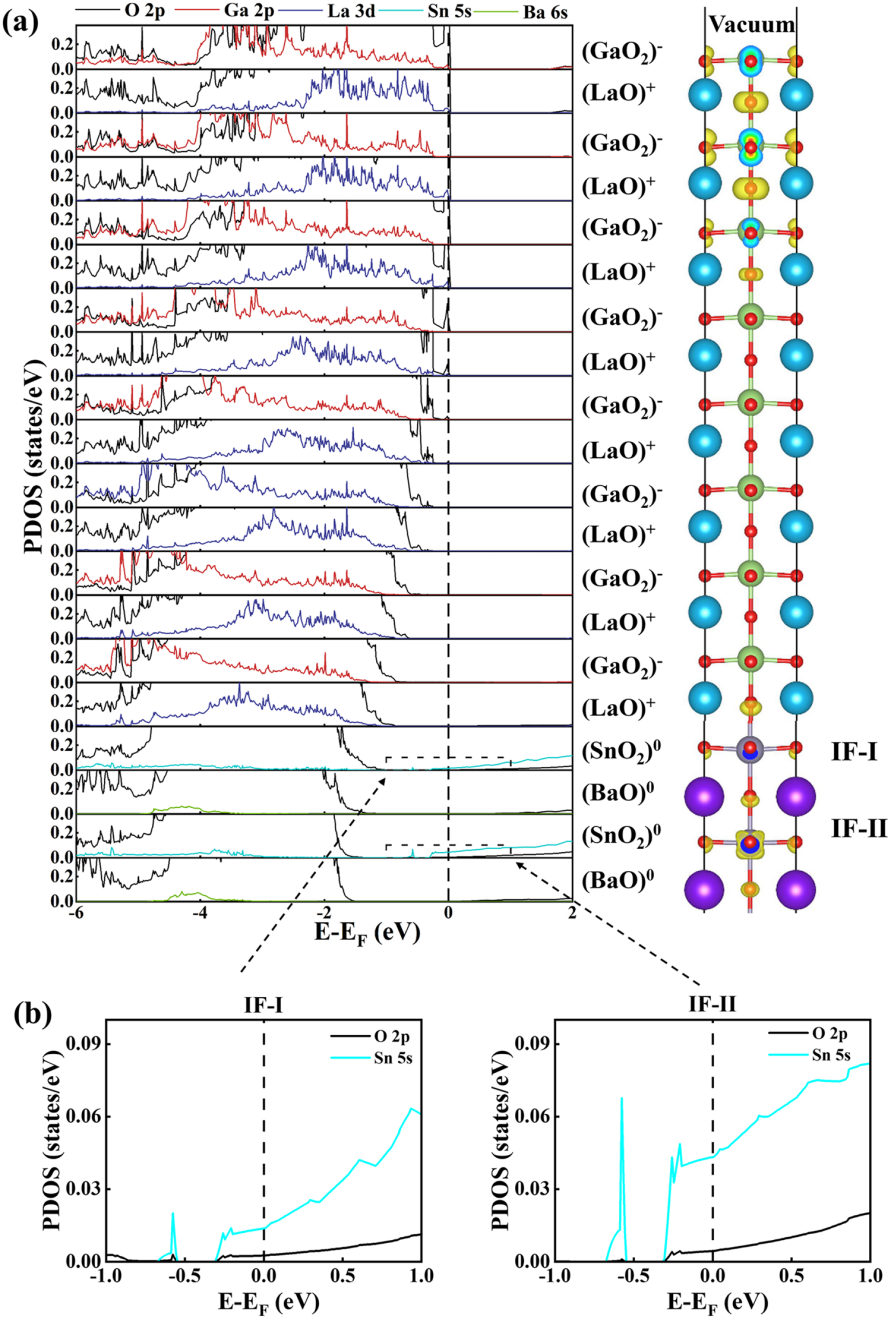
Calculated layer-resolved partial DOS for the *n*-type (LaGaO_3_)_3_/BaSnO_3_ HS model along with the charge density projected on bands forming the 2DEG. The isovalue of 1.1$\times$ 10^−4^ e/bohr^3^ is used to produce the charge density plots.

To further understand the origin and charge transfer of metallic electronic states in the *n*-type (LaGaO_3_)_*m*_/BaSnO_3_ HS system, We calculated the layer-resolved partial DOS for *n*-type (LaGaO_3_)_*m*_/BaSnO_3_ HS system at m=2, 4 and 8, as shown in Figs. 2, S1 in the supporting information and Fig. 3, respectively. To directly observe the contribution of each layer to the metallic states, we calculated the charge density projected on metallic bands for each model. For the convenience of discussion, the first and second layers of SnO_2_ are defined as IF-I and IF-II, respectively. For the (LaGaO_3_)_2_/BaSnO_3_ HS system in Fig. 2, the layer-resolved partial DOS and the charge density show that the fermi level does not cross the conduction bands and valence bands. Then there are no metallic states exist in the IF-I and IF-II layers, and the HS system shows insulating characteristic. These phenomena can be clearly seen in the enlarged view of IF-I and IF-II layers in Fig. 2b. Since the polarization strength of LaGaO_3_ film in (LaGaO_3_)_2_/BaSnO_3_ HS system is strong enough to make the electrons transfer from the (LaO)^+^ layer to (GaO_2_)^−^ layer, neutralizing the holes in (GaO_2_)^−^ layer. Then there exist no holes on the surface and no electrons at the interface. The polar discontinuity in the *n*-type (LaO)^+^/(SnO_2_)^0^ interface is offset by strong polarization in LaGaO_3_ film, which is similar to the LaAlO_3_/SrTiO_3_ HS system^31^.

For the (LaGaO_3_)_8_/BaSnO_3_ HS system in Fig. 3, the fermi level passes through the valence bands of the surface (GaO_2_)^−^ layer, showing *p*-type conducting states. While the interface indicates *n*-type conducting states from the interfacial (SnO_2_)^0^ layer. Then the overlap of these states make the (LaGaO_3_)_8_/BaSnO_3_ HS system show metallic property in Fig. 1. The O2*p* states of (LaO)^+^ and (GaO_2_)^−^ layers significantly shift toward higher energy with the layers move from the interfacial (LaO)^+^ layer to the surface (GaO_2_)^−^layer, presenting the electrostatic potential in the LaGaO_3_ film. This is because that the LaGaO_3_ film in the (LaGaO_3_)_8_/BaSnO_3_ HS model exhibits a weaker polarization than that in the (LaGaO_3_)_2_/BaSnO_3_ HS model, which is not enough to offset the polarity discontinuity between LaGaO_3_ and BaSnO_3_. Thus, there exist *p*-type conducting states from O 2*p* orbitals on the LaGaO_3_ surface. In short, the (LaGaO_3_)_8_/BaSnO_3_ HS system present metallic properties with the *p*-type conducting states from O 2*p* orbitals on the surface and the *n*-type conducting states from Sn 5*s* orbitals at the interface.

**Figure 4:**
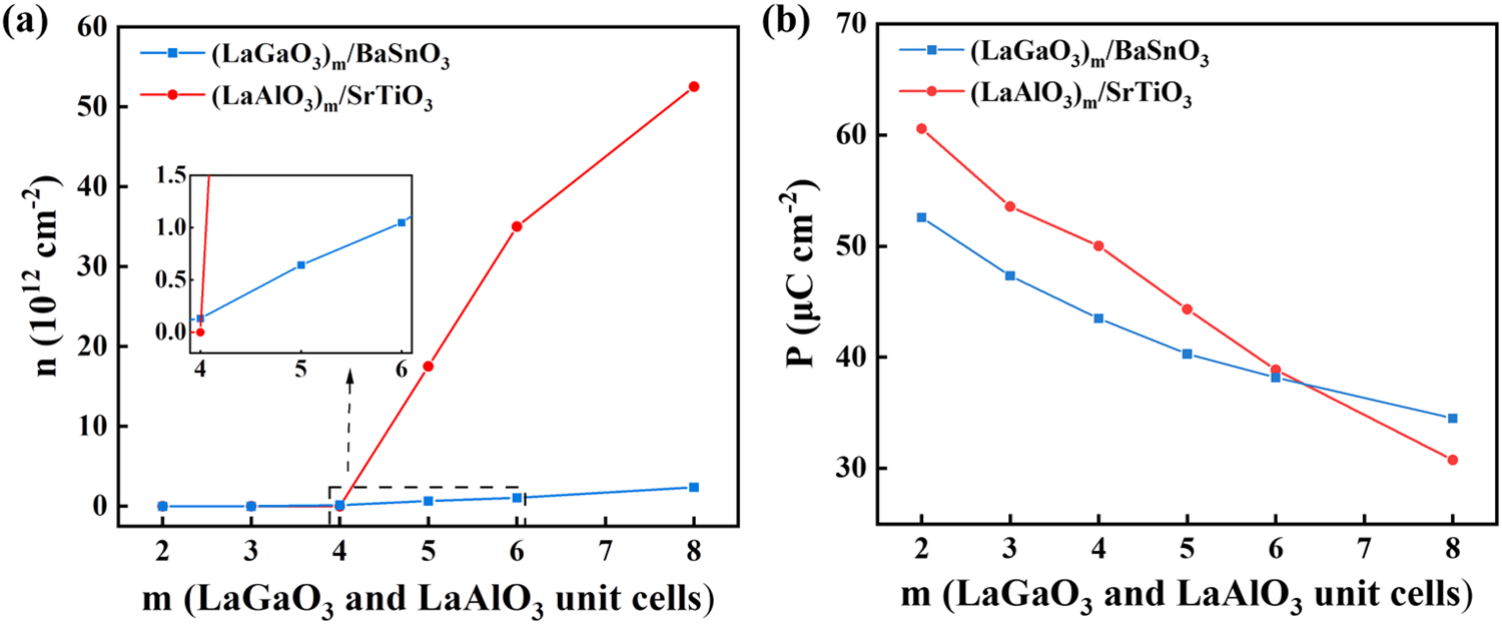
(**a**) Calculated interfacial charge carrier density (*n*) and (**b**) Polarization strength (P) in LaGaO_3_ (LaAlO_3_) film for the *n*-type LaGaO_3_/BaSnO_3_ (LaAlO_3_/SrTiO_3_) HS system as a function of LaGaO_3_ (LaAlO_3_) unit cells. The inset is the locally enlarged view of interfacial charge carrier density for LaGaO_3_/BaSnO_3_ HS system.

To clearly present the transition from the insulating characteristic to the metallic property of LaGaO_3_/BaSnO_3_ HS system, we also calculated the layer-resolved partial DOS for the *n*-type (LaGaO_3_)_4_/BaSnO_3_ HS model along with the charge density projected on bands forming the 2DEG, shown in Fig. S1 in the supporting information. All the characteristics of (LaGaO_3_)_4_/BaSnO_3_ HS model are consistent with that of (LaGaO_3_)_8_/BaSnO_3_ HS system, except that the surface and interface metallic states are less. To quantify the change of interfacial electron concentration of the (LaGaO_3_)_*m*_/BaSnO_3_ HS system with the LaGaO_3_ film thickness, we also calculated their interfacial electron concentration by integrating the partial DOS of the Sn 5*s* orbitals near the fermi level from the interfacial IF-I and IF-II (SnO_2_)^0^ layer divided by the interfacial area, shown in Fig. 4a. As a comparison, the interfacial carrier concentration of (LaAlO_3_)_*m*_/SrTiO_3_ HS system are also calculated in Fig. 4a, which is about 2−6$\times$10^13^ cm^−2^ at $m\ge 5$. For (LaGaO_3_)_*m*_/BaSnO_3_ HS system, at $m\le 3$, the interfacial electron concentration is zero; at $m\ge 4$, as m increases, the interfacial electron concentration increases. Similar to LaAlO_3_/SrTiO_3_ HS system, a sudden increase of interfacial electron concentration in LaGaO_3_/BaSnO_3_ HS system occur from m=4 to m=5. But he values of electron concentration are on the order of 10^12^ cm^−3^, which is about an order of magnitude smaller than that in the corresponding (LaAlO_3_)_*m*_/SrTiO_3_ HS system. This is because that the Sn 5*s* orbitals are more dispersive and poorly localized for electrons than the Ti 3*d* orbitals. This phenomenon is unfavorable to the practical application of 2DEG. Therefore, we indicate that the localization of interfacial electrons for the BaSnO_3_-based HS system can be improved by doping the elements who have *d* orbitals. This method has already been used to modify the electrical properties of BaSnO_3_ film. For example, Bing Li *etal*.^32^ prepared Nb doped BaSnO_3_ films by pulsed laser deposition, showing that the BaNb_0.05_Sn_0.95_O_3_ film simultaneously has a high electron mobility of 19.65 cm^2^V^−1^s^−1^and electron density of 6.59 $\times$10^20^ cm^−3^, which is beneficial to its application in optoelectronic devices.

To analyze the change of interfacial 2DEG for LaGaO_3_/BaSnO_3_ HS system with different LaGaO_3_ film thickness, the average polarization strength in the LaGaO_3_ film was calculated by the following equation^33,34^:

1 $$\begin{aligned} P=\frac{e}{\Omega }\sum _{i=1}^N Z_i^*\cdot \delta _{z_i} \end{aligned}$$

where $\Omega$ is the total volume of the LaGaO_3_ film, N is the total number of atoms in the unit cell, Z_i_^*^ is the Born effective charge of each atom, and $\delta _{z_i}$ is the relative displacement of the ith atom in the HS system. The relative displacement $\delta _{z_i}$ of La(Ga) atoms with respect to the oxygen atom in the same LaO and GaO_2_ layers is calculated as $\delta _{z_{La/Ga}} = z_{La/Ga} - z_O$. Our calculated Born effective charge Z_i_^*^ for La and Ga atoms are 4.03 and 3.34, respectively.

The calculated polarization strength in the LaGaO_3_ film of *n*-type (LaGaO_3_)_*m*_/BaSnO_3_ HS system with different LaGaO_3_ thickness are shown in Fig. 4b. As the LaGaO_3_ film thickness increases from 2 to 8, the average polarization strength in the LaGaO_3_ film decreases from 52.59 $\mu$C$\cdot$ cm^−2^ to 34.52 $\mu$C$\cdot$ cm^−2^ , indicating that the electrostatic force drives the electron from (LaO)^+^ layer to (GaO_2_)^−^ layer is weaken, leading to the increase of interfacial electron density. Combined with the total DOS diagram in Fig. 1, it can be concluded that the critical polarization strength of insulator-to-metal transition for LaGaO_3_/BaSnO_3_ HS system is 43.50 $\mu$C$\cdot$ cm^−2^ (polarization strength at m=4), which is lower than that of LaAlO_3_/SrTiO_3_ HS system with the value of 50.03 $\mu$C$\cdot$ cm^−2^ .

### 2DEG in the strained LaGaO_3_/BaSnO_3_ HS system

**Figure 5:**
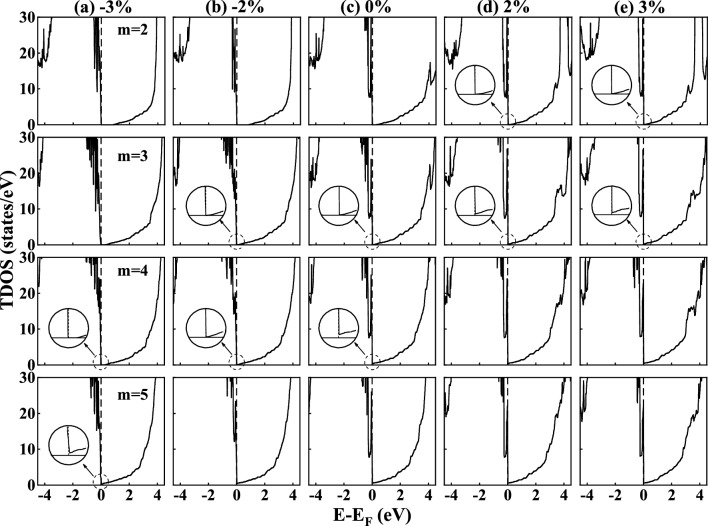
Calculated total DOS for the *n*-type (LaGaO_3_)_*m*_/BaSnO_3_ (*m* =2, 3, 4, and 5) HS models with different strains. (**a**) − 3%, (**b**) − 2%, (**c**) 0%, (**d**) 2% and (**e**) 3%. The insets are the enlarged view of the DOS near fermi level for models.

Based on the discussions above, we know that the critical thickness of insulator-to-metal transition for the unstrained (LaGaO_3_)_*m*_/BaSnO_3_ HS system is 4 unit cells. The formation of 2DEG at the interface is strongly related to the distortion of LaGaO_3_ film. To study the changes of interfacial electronic states induced by this distortion, we calculated the total DOS of (LaGaO_3_)_*m*_/BaSnO_3_ (m=2, 3, 4, and 5) HS models with different biaxial strains of −3%, −2%, 2%, and 3%, as shown in Fig. 5. The “$+$” and “−” signs indicate tensile and compressive strains, respectively. Our calculated total DOS shows that all the (LaGaO_3_)_2_/BaSnO_3_ HS models with − 3%, − 2%, 0, 2%, and 3% strains show semiconducting characteristics (first row in Fig. 5). While the (LaGaO_3_)_3_/BaSnO_3_ HS model with − 3%, − 2%, and 0 strains and the (LaGaO_3_)_4_/BaSnO_3_ HS model with −3% strain also show a similar semiconducting behavior. With the strain from −3% to 3%, the band gap of (LaGaO_3_)_4_/BaSnO_3_ HS model decreases and vanishes. A small number of states present at the fermi level for the models with 0, 2% and 3% strains, thus exhibiting weak metallicity. All the (LaGaO_3_)_5_/BaSnO_3_ HS models exhibit metallic properties and more states arise near the fermi level with the strain from compressive to tensile. In short, we can obtain the following conclusions: (1) For the unstrained (LaGaO_3_)_5_/BaSnO_3_ HS system, 4 unit cells of LaGaO_3_ is the minimum critical thickness to obtain a 2DEG; (2) For the −3%-biaxially-strained HS system, a critical thickness of 5 unit cells of LaGaO_3_ is required, while 3 unit cells are the minimum requirement to form a 2DEG for the 3%-biaxially-strained HS system. These results present biaxial strains on the BaSnO_3_ substrate has a significant impact on the critical thickness of LaGaO_3_ film for forming 2DEG.

To exhibit the distribution of 2DEG for the *n*-type (LaGaO_3_)_*m*_/BaSnO_3_ HS models with various biaxial strains, we further plotted the partial density states of Sn 5*s* orbitals in IF-I and IF-II layers for (LaGaO_3_)_4_/BaSnO_3_ HS system with the biaxial strains of − 3%, 0, and 3% in Fig. S2 in the supporting information. The fermi level of the (LaGaO_3_)_4_/BaSnO_3_ HS system with −3% strain does not cross the Sn 5*s* orbitals, exhibiting an insulating property. But for the unstrained and 3%-strained (LaGaO_3_)_4_/BaSnO_3_ HS models, the fermi level crosses the Sn 5*s* orbitals, showing an metallic property. Moreover, the 3%-strained (LaGaO_3_)_4_/BaSnO_3_ HS system has a higher concentration of electronic states near the fermi level compared to the unstrained HS system.

**Figure 6:**
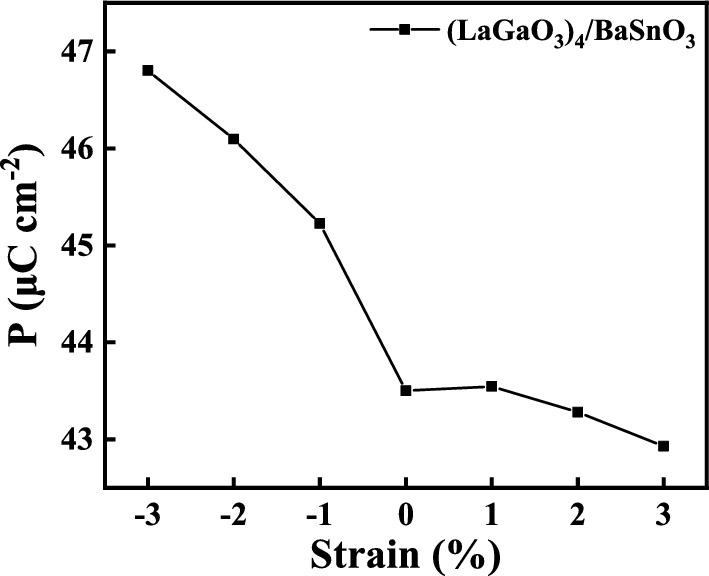
Calculated average polarization strength (P) in the LaGaO_3_ film for *n*-type (LaGaO_3_)_4_/BaSnO_3_ HS models with different biaxial strains.

To further understand the change of electronic states at the interface, the polarization strength of (LaGaO_3_)_4_/BaSnO_3_ HS system with the biaxial strain from − 3 to 3% is calculated, as shown in Fig. 6. The average polarization strength of (LaGaO_3_)_4_/BaSnO_3_ HS system decreases with the biaxial strains from − 3 to 3%. In Fig. 4, we know that the critical polarization strength of unstrained LaGaO_3_/BaSnO_3_ HS system is 43.50 $\mu$C$\cdot$ cm^−2^ corresponding to the values of HS system with 4-unit-cells thickness of LaGaO_3_ film. The average polarization strength of compressively-strained (LaGaO_3_)_4_/BaSnO_3_ HS system is larger than that of the unstrained HS system. The electrostatic force prevent electron transfer from (LaO)^+^ layer to (GaO_2_)^−^ layer is strengthen, resulting in insulating behavior for the compressively-strained (LaGaO_3_)_4_/BaSnO_3_ HS system. On the opposite, the average polarization strength of tensilely-strained HS system is smaller than that of the unstrained HS system. Then the electrostatic force present the electron from (LaO)^+^ layer to (GaO_2_)^−^ layer is weaken, thus the (LaGaO_3_)_4_/BaSnO_3_ HS system under biaxial tensile strain present a metallic property. Overall, the polarization strength of (LaGaO_3_)_4_/BaSnO_3_ HS system is strongly influenced by the biaxial strain, which determining the critical thickness of insulator-to-metal transition.

**Figure 7:**
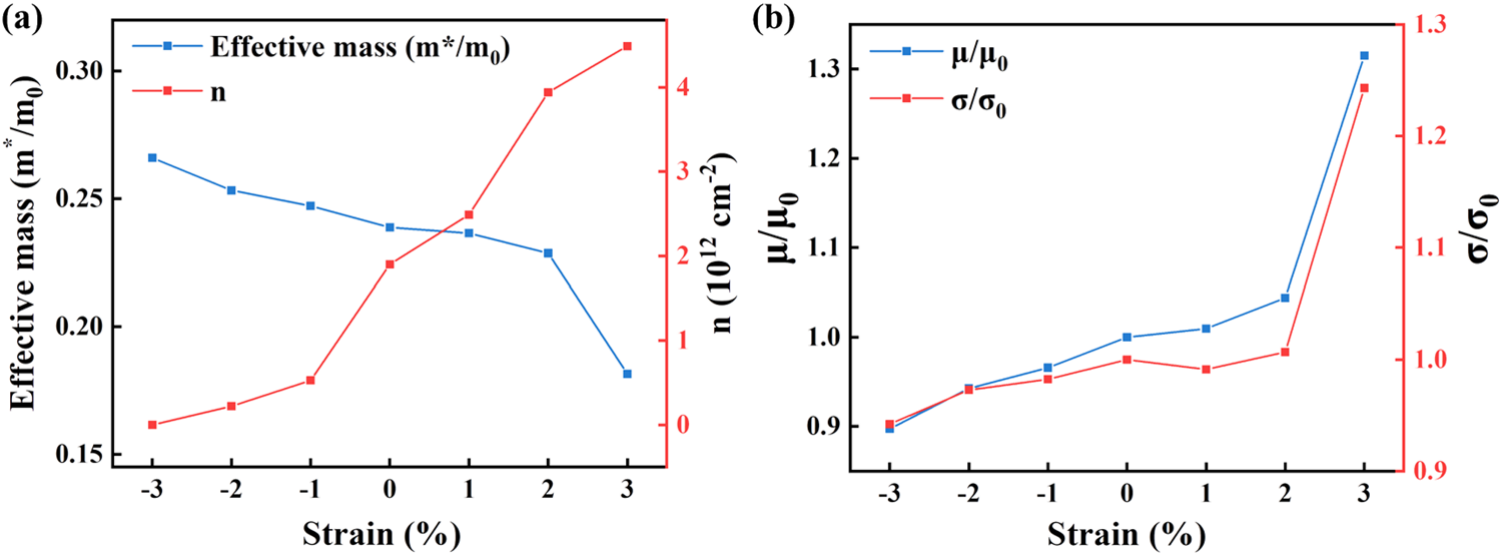
(**a**) The electron effective mass (m^+^/m_0_) and interfacial electron density (*n*), (**b**) The normalized electron mobility ($\mu$/$\mu _0$) and normalized electrical conductivity ($\sigma$/$\sigma _0$) for the (LaGaO_3_)_4_/BaSnO_3_ HS model under different biaxial strains.

Next, we calculated the electronic band structures for the strained (LaGaO_3_)_4_/BaSnO_3_ HS system along the path M-$\Gamma$-X of the interfacial Brillouin zone compared to the unstrained model, shown in Fig. S3 in the supporting information. Some electronic states reside below the fermi level for the unstrained and tensilely-strained (LaGaO_3_)_4_/BaSnO_3_ HS system, indicating metallic properties. While the compressively-strained HS system exhibit an insulating property. Further, we calculated the electron effective mass (m^+^/m_0_) for the minimum conduction bands along the $\Gamma$-*X* and $\Gamma$-*M* directions, in which $m_0$ is the electron effective mass for free electron. The electron effective mass m^+^ was calculated using the parabolic approximation by the following formula^35^:

2 $$\begin{aligned} \frac{1}{m^*} = \frac{1}{\hbar ^2} \frac{\partial ^2E_{CB}}{\partial \kappa ^2} \end{aligned}$$

where $\hbar$ is the reduced plank constant, $\kappa$ is the corresponding wave vector of the conduction bands, and E_CB_ is the energy of the minimum conduction band. For the unstrained (LaGaO_3_)_4_/BaSnO_3_ HS system, the calculated electron effective mass is 0.24m_0_, which is in good agreement with that of BaSnO_3_ bulk^36^. As the biaxial strain changes from −3% to 3%, the electron effective mass decreases from 0.27m_0_ to 0.18m_0_. That is, the biaxial compressive strains harm the electron migration characteristic, and the tensile strains play the opposite role. What’s more, we calculated the charge carrier density by integrating the layer-resolved partial DOSs for the conducting states below the fermi level from the interfacial IF-I and IF-II (SnO_2_)^0^ layer, see Fig. 7a. The HS systems with biaxial strains from −3 to 3% exhibit an increasing charge density from 0 to 4.49$\times$10^12^cm^−2^, which provides evidence that the biaxial compressive strain suppresses the production of 2DEG, and the biaxial tensile strain promotes the formation of 2DEG at the interface.

Besides the interfacial charge density, the electron mobility is also an important factor in determining the interfacial conductivity of the 2DEG. We calculated the normalized electron mobility ( $\mu$/ $\mu _0$) and normalized electrical conductivity ( $\sigma$/ $\sigma _0$) for (LaGaO_3_)_4_/BaSnO_3_ HS system under different biaxial strains, see Fig. 7b. $\mu _0$ and $\sigma _0$ refer to the electron mobility and electrical conductivity of the unstrained (LaGaO_3_)_4_/BaSnO_3_ HS system, respectively. The following two Eqs. (3) and (4) were used^37^:

3 $$\begin{aligned} \mu= & {} e<\tau >/m^* \end{aligned}$$

4 $$\begin{aligned} \sigma= & {} ne\mu \end{aligned}$$

where *e*, $<\tau >$, $m^*$ , *n*, $\mu$, and $\sigma$ are the elementary charge, average scattering time, electron effective mass, interfacial electron density, electron mobility, and electrical conductivity, respectively. The scattering time $\tau$ is determined by all the scattering events, i.e. impurity scattering, electron-phonon scattering and electron-electron scattering. The inverse of $\tau$ can be described as the sum of rates associated with all the scattering mechanisms according to Matthiessen’s rule^38^. It has been extremely challenging to calculate the scattering time $\tau$ due to the complicated scattering mechanisms. One common and simplified approach is to treat $\tau$ as a constant, which has been validated in prior studies^35,39,40^ and used in this work. The electron mobility and the electrical conductivity of (LaGaO_3_)_4_/BaSnO_3_ HS system with different biaxial strains are presented in Fig. 7b. As the biaxial strain changes from −3 to 3%, the value of $\mu$/$\mu _0$ and $\sigma$/$\sigma _0$ increases. Compared to the unstrained HS system, the $\mu$/$\mu _0$ and $\sigma$/$\sigma _0$ for compressively-strained (LaGaO_3_)_4_/BaSnO_3_ HS system show lower, while for tensilely-strained HS system present higher.

## Discussion

In conclusion, the insulator-to-metal transition critical thickness and electrical properties of unstrained and strained LaGaO_3_/BaSnO_3_ HS slab systems are studied using density functional theory calculations. The results show that a critical thickness of 4 unit cells for LaGaO_3_ film is required for forming 2DEG in the unstrained LaGaO_3_/BaSnO_3_ HS system, while the critical thickness of LaGaO_3_ film increases up to 5 unit cells in – 3%-biaxially-strained HS system and decreases to 3 unit cells in 3%-biaxially-strained HS system. These results are originated from that the biaxial strain along *ab* plane on the BaSnO_3_ substrate can significantly affects the polarization strength in the LaGaO_3_ film. We also find that the biaxial tensile strain can considerably increase the interfacial charge carrier density, electron mobility and electrical conductivity, while the biaxial compressive strain shows the opposite effect. In short, the interfacial electrical properties of 2DEG in LaGaO_3_/BaSnO_3_ HS system can be optimized by applying a tensile strain on the BaSnO_3_ substrate along the *ab*-plane.

## Methods

In this work, all the density functional theory (DFT)^41^ calculations were carried out using the Vienna Ab initio Simulation Package (VASP)^42,43^. The projector augmented-wave (PAW) potentials were applied for electron-ion interactions^44^. The generalized gradient approximation (GGA) parameterized by Perdew-Burke-Ernzerhof (PBE) with the on-site Coulomb interaction approach (GGA+U) was employed for the exchange-correlation functional^45^. Since the electronic properties of perovskite oxides are sensitive to the U value of transition metal ions. An empirical U value of 7.5 eV was used to describe La 4*f* orbitals. A cutoff energy of 450 eV was used for the plane-wave basis set, and k-space grids of 10$\times$10$\times$1 within the monkhorst-pack scheme^46^ were employed to converge the total energy. The electronic self-consistency calculation was assumed for a total energy convergence of less than 10^−5^ eV. All the atomic position were optimized until the interatomic forces smaller than 0.03 eV Å^−1^.

BaSnO_3_ crystallizes in a cubic phase with a space number of 211 (Pm$\bar{3}$m) at RT^47^, while LaGaO_3_ exhibits an orthogonal perovskite structure. A symmetric sandwich-type structural model, (LaGaO_3_)_*m*_/(BaSnO_3_)_12.5_/(LaGaO_3_)_*m*_, was built to model the *n*-type (LaO)^+^/(SnO_2_)^0^ interface by adding different thickness of LaGaO_3_ film on the SnO_2_-terminated BaSnO_3_ with the thickness of 12.5 unit cells. A vacuum layer of 20 Å was added on the GaO_2_-terminated LaGaO_3_ films to avoid the dipole-dipole interaction between the periodic slabs. To model the epitaxial growth process, all the ions were fully relaxed with fixed lattice parameters along the *ab* plane. The value of *m* was set from 2 to 8 to figure out the formation mechanism of 2DEG at the interface. The lattice parameter was changed to simulate various strains from – 3 to 3%, at intervals of 1% in the *ab* plane.

## Supplementary Information

Supplementary Information.

## Data Availability

The data that support the findings of this study are not openly available, but are available from the corresponding author upon reasonable request. If necessary, please contact the corresponding author: wangyqyyxf@sina.com.
