# Supplementary material for: Density functional theory study on the formation mechanism and electrical properties of two-dimensional electron gas in biaxial-strained LaGaO3/BaSnO3 heterostructure
Source: Sci Rep. 2024 May 4;14:10259. doi: 10.1038/s41598-024-60893-y (PMC11636876; doi:10.1038/s41598-024-60893-y)
Supplement: Supplementary file 1 — Supplementary Information. [file 41598_2024_60893_MOESM1_ESM.docx]

**Supporting Information**

**Density functional theory study on the formation mechanism and electrical properties of two-dimensional electron gas in biaxial-strained LaGaO_3_/BaSnO_3_ heterostructure**

Yuling Li^1^, Yuxi Huang^1^, Xiaohua Liu^1^, Yaqin Wang^1,2,*^, and Le Yuan^1^

*^1^Xihua University, Key Laboratory of Fluid and Power Machinery, School of Material Science and Engineering, Chengdu, 610039, P. R. China*

*^2^University of Electronic Science and Technology of China, State Key Laboratory of Electronic Thin Films and Integrated Devices, Chengdu, 610054, P. R. China*

*E-mail: wangyqyyxf@sina.com

**Table S1** Experimental and calculated structural parameters and band gaps within GGA+U method of cubic LaGaO_3_ and BaSnO_3_.

| Compound | experiment | |  | DFT | |
| --- | --- | --- | --- | --- | --- |
|  | a（Å） | E_g_(eV) |  | a（Å） | E_g_(eV) |
| LaGaO_3_ | 3.860^1^ | 4.4^2^ |  | 3.939 | 3.668 |
| BaSnO_3_ | 4.115^3^ | 3.1^4^ |  | 4.186 | 2.208 |

*、*

**
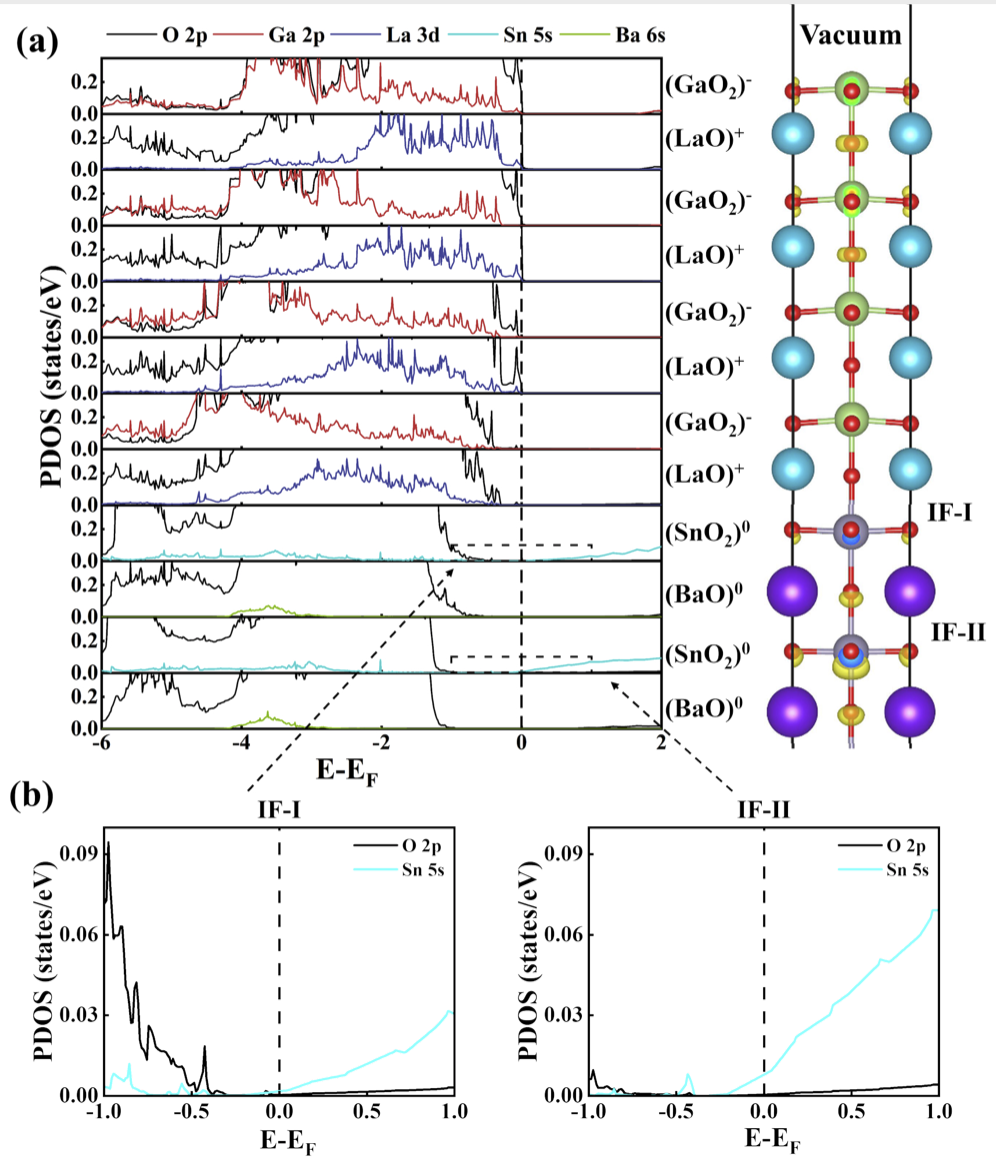
**

Figure S1 Calculated layer-resolved partial density of states (DOS) for the *n*-type (LaGaO_3_)_4_/BaSnO_3_ HS model along with the charge density projected on bands forming the 2DEG. The isovalue of 1.1×10^-4^ e/bohr^3^ is used to produce the charge density plots.

**
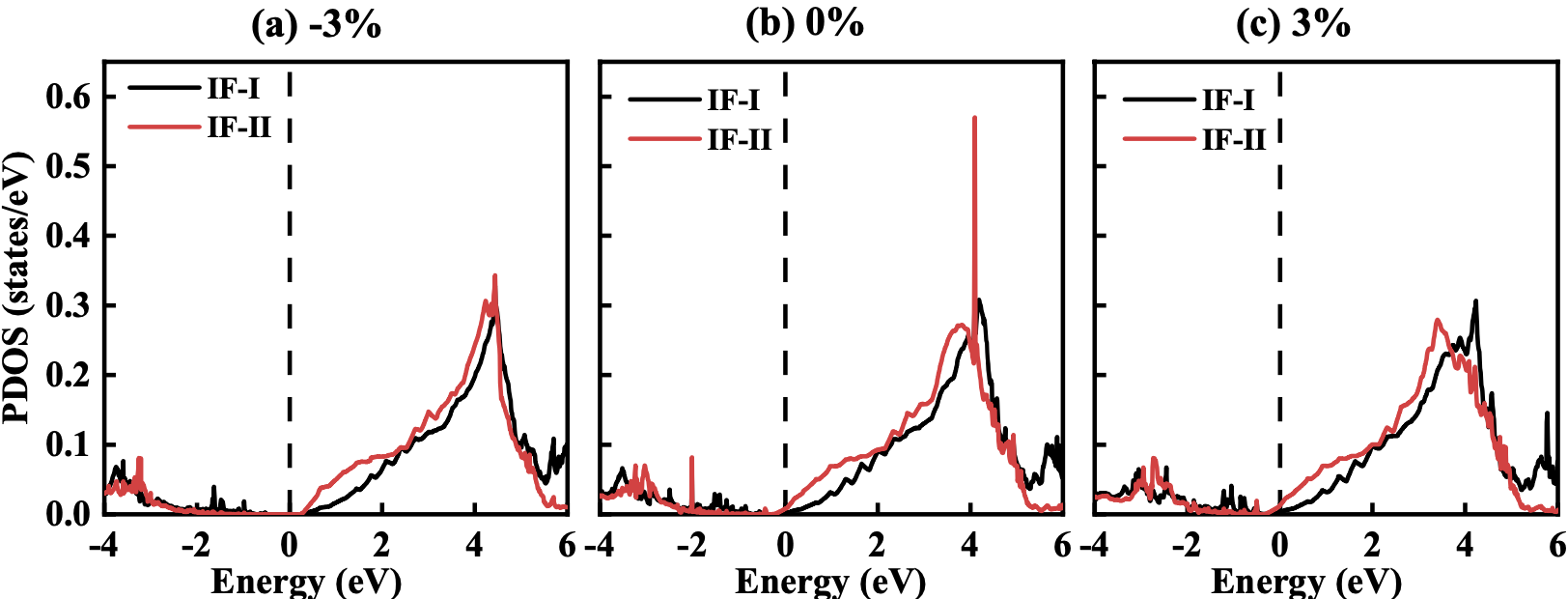
**

Figure S2 Calculated partial DOS of Sn 5*s* orbitals in IF-I and IF-II layers for (LaGaO_3_)_4_/BaSnO_3_ HS system with different biaxial strains. (a) -3%, (b) 0, (c) 3%.


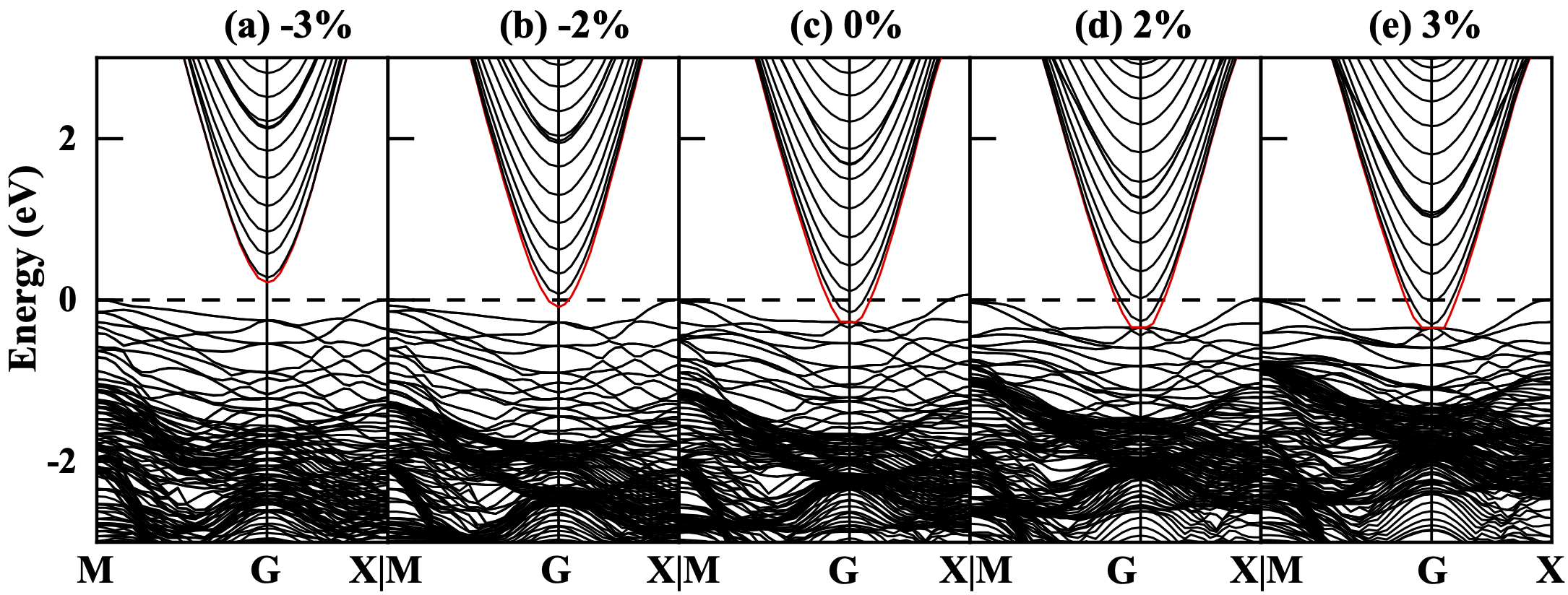


Figure S3 Calculated electronic band structures of (LaGaO_3_)_4_/BaSnO_3_ HS models under different biaxial strains. (a) -3%; (b) -2%; (c) 0; (d) 2%; (e) 3%

**References**

1. Perna, P., et al., Conducting interfaces between band insulating oxides: The LaGaO_3_/SrTiO_3_ heterostructure. *Appl. Phys. Lett*. **97**. 152111. DOI: 10.1063/1.3496440 (2010).
2. Kiyonori Ogisu, et al., Electronic Band Structures and Photochemical Properties of La-Ga-based Oxysulfides. *J. Phys. Chem. C*. **112**. 11978--11984. DOI: 10.1021/jp802153t (2008).
3. Wadekar, P.V., et al., Improved electrical mobility in highly epitaxial La: BaSnO_3_ films on SmScO_3_ (110) substrates. *Appl. Phys. Lett*. **105**. 052104 DOI: 10.1063/1.4891816 (2014).
4. Zhang, W., J. Tang, and J. Ye, Structural, photocatalytic, and photophysical properties of perovskite MSnO_3_ (M = Ca, Sr, and Ba) photocatalysts. *J. Mater. Res.* **22**. 1859-1871. DOI: 10.1557/jmr.2007.0259 (2011).
